# Sorption: A Statistical Thermodynamic Fluctuation Theory

**DOI:** 10.1021/acs.langmuir.1c00742

**Published:** 2021-06-14

**Authors:** Seishi Shimizu, Nobuyuki Matubayasi

**Affiliations:** † York Structural Biology Laboratory, Department of Chemistry, University of York, Heslington, York YO10 5DD, United Kingdom; ‡ Division of Chemical Engineering, Graduate School of Engineering Science, Osaka University, Toyonaka 560-8531, Osaka, Japan

## Abstract

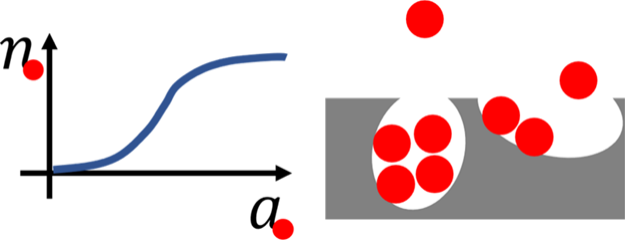

Can the sorption mechanism be proven by fitting an isotherm model to an experiment? Such a question arises because (i) multiple isotherm models, with different assumptions on sorption mechanisms, often fit an experimental isotherm equally well, (ii) some isotherm models [such as Brunauer–Emmett–Teller (BET) and Guggenheim–Anderson–de Boer (GAB)] fit experimental isotherms that do not satisfy the underlying assumptions of the model, and (iii) some isotherms (such as Oswin and Peleg) are empirical equations that do not have a well-defined basis on sorption mechanisms. To overcome these difficulties, we propose a universal route of elucidating the sorption mechanism directly from an experimental isotherm, without an isotherm model, based on the statistical thermodynamic fluctuation theory. We have shown that how sorbate–sorbate interaction depends on activity is the key to understanding the sorption mechanism. Without assuming adsorption sites and planar layers, an isotherm can be derived, which contains the Langmuir, BET, and GAB models as its special cases. We have constructed a universal approach applicable to adsorption and absorption, solid and liquid sorbents, and vapor and liquid sorbates and demonstrated its efficacy using the humidity sorption isotherm of sucrose from both the solid and liquid sides.

## Introduction

Sorption isotherms play an important role in all aspects of our daily lives from food,^1−3^ clothing,^4^ and building,^5−7^ as well as in diverse scientific areas, such as biomolecules and colloids,^8^ activated carbons,^9,10^ nanoparticles,^11^ and aerosols.^12^ Understanding the molecular interactions underlying an isotherm is crucial.

However, there are more than 80 different isotherm models published so far, each lying on a spectrum between empirical and physical.^13−18^ The empirical models (such as the Oswin^19^ and Peleg^20,21^) do not have a well-defined physical basis, and despite their practical value, insights on the adsorption mechanism may not be gained by fitting such a model to an experimental isotherm. The physical models [such as the Langmuir,^22^ Brunauer–Emmett–Teller (BET),^23,24^ and Guggenheim–Anderson–de Boer (GAB)^25−27^] are founded on assumed adsorption mechanisms, such as adsorption sites, layers, their numbers, and binding constants.^13−18^ However, some of the most popular physical models have been applied routinely beyond their basic assumptions and premises.^20^ Doubts have been raised whether the goodness of fit is a sufficient criterion to judge the correctness of a sorption mechanism because different types of models can fit an experimental isotherm equally well.^20,28^ In the face of these difficulties, the objective of this paper is threefold:

I to establish a universal sorption theory applicable to adsorption and absorption, solid and liquid sorbents, and vapor and liquid sorbates,
II to reveal the molecular interactions underlying an experimental isotherm as well as an isotherm model, and
III to clarify the similarity and difference between sorption and solvation.

These objectives have immediate ramifications to the use of isotherm models in the study of sorption. We will demonstrate that (i) the actual interpretation of the parameters calculated from an isotherm model may be different from what they claim to be and that (ii) the sorption mechanism can be clarified directly from an experimental isotherm without relying on isotherm models and their assumptions.

A universal approach to sorption must be applicable across the traditional classifications and categories, such as adsorption versus absorption^29^ and sorbents versus solvents.^30,31^ Such a classification is founded on experimental observations and the reality of the system. Yet, many difficulties arise across these categories. For example, the routine application of adsorption models^23−27,32−34^ to absorption phenomena^20^ and identifying “sorbate structures” with “solvent structures” in the study of “water structures”^35,36^ may have led to confusion. In the following, we shall present a brief sketch of these difficulties to show that a unified theory across the classification boundary is indispensable for overcoming these difficulties.

### Adsorption versus Absorption

The BET,^23,24^ GAB,^25−27^ and Frenkel–Halsey–Hill (FHH)^32−34^ models were proposed to explain multilayer adsorption on planar surfaces. These models have been applied to fit sorption isotherms of far more complex systems (such as moisture on wood,^6,7^ powders,^37^ aerosol,^12^ rock,^38^ and food^1−3,39−41^); difficulties have arisen when assuming that these complex sorption phenomena lead to multilayer adsorption onto a plane. Recognizing the nonplanar nature of sorbents at the core of these difficulties, the fractal nature of surfaces has been taken into account for the multilayer models such as the BET^23,24^ and FHH models.^14−18^ However, how the “fractal dimension” *D* has been introduced is different from one model to another,^42,43^ and different values of *D* depending on the range of sorbate vapor pressure^44,45^ and even the values of *D* exceeding those of the embedding environment (i.e., 3) have sometimes been reported.^18,46−48^ Furthermore, doubts have been raised on the foundations of the BET–GAB and FHH models themselves. The FHH model and its fractal generalization^14−16^ are based on an assumed distance variation of the “adsorption potential”,^49^ which, according to Dubinin, has “in itself no physical meaning for adsorption in micropores”.^50^ The monolayer assumption, one of the key assumptions of the BET–GAB models, has also been questioned.^20^ For example, the water sorption isotherm on starch granules showed no dependence on the BET surface area;^51^ discrepancies in calculated monolayer adsorption arise when different adsorption models were adopted;^52^ and the same isotherm model (fractal BET) can fit different behaviors arising from the variation of cellulose crystallinity, that is, water adsorption without swelling or absorption with swelling.^53^ These difficulties necessitate a universal theory that applies to both adsorption and absorption, regardless of surface geometry such as porosity.

### Sorbent versus Solvent

The uptake of moisture or gas by liquids and solutions has been studied for a long time,^29,54,55^ with important applications such as CO_2_ capture^56^ and moisture sorption in liquid food and drinks.^1−3,35,39−41^ However, difficulties have arisen whenever solvation in the solution phase was confused with adsorption onto a solid surface due to an apparent similarity between solvation and adsorption.^30,36,57^ For example, the key contribution to the Norrish constant, presumed to represent the “water structure” in liquid food, turned out to contain significant contributions from solute–solute interaction.^35^ Moreover, the osmotic stress technique,^58,59^ which was founded on an apparent analogy between preferential solvation and the Gibbs adsorption isotherm, misattributed the exclusion of osmolytes from the protein surface to protein hydration increase.^30,36,57^ Such confusion stems from an apparent similarity between sorption and solvation, which has been invoked for a long time.^60,61^ In this context, the extension of solution-phase fluctuation to adsorption by Zimm^60^ and Zimm and Lundberg,^61^ and its subsequent applications beyond liquid sorbents,^62−66^ must be re-examined. For these reasons, a universal theory of sorption, which applies to solid and liquid sorbents alike, is needed.

### Sorbent Transition

A hygroscopic powder sorbent, after a critical relative humidity called the deliquescence point, dissolves in water.^67−72^ A sharp transition in the isotherm is a signature of the deliquescence transition.^71,72^ From the solution side, the addition of more solutes (such as sucrose) into a liquid sorbent solidifies the system. These transitions accompany an overall change in the physical state of the sorbent and an overall change in molecular mobility manifested as the change of plasticity and viscosity, as well as caking.^67−72^ Even though these properties are dynamic rather than thermodynamic, the sorption isotherm is still considered to be an important physical property; these common observations are rationalized often by assuming that “water in amorphous solids can exist in both a “bound” and a “solvent-like” state, with, perhaps, two types of “bound” states”.^69^ Consequently, many adsorption isotherm models, which focus exclusively on the “bound water”, cannot say anything about the “solvent-like water” which is often invoked in interpretation. Since sorption isotherms play a crucial role^73^ in understanding how manufacturing conditions, such as granular size, tablet compression, crystallinity, or coating, affect the transitions,^51,74−78^ a universal theory of sorption, which encompasses the different degrees of sorbent mobility, is necessary.

Thus, our goal is to develop a universal theory of sorption that can be used for adsorption and absorption and solid and liquid adsorbents alike, without any limitations on surface geometry imposed by model assumptions or (semi-)empirical formulae.^49,79^ Our foundation is the principles of statistical thermodynamics.^80,81^ We have previously published a rigorous approach to solvation in multiple-component solutions^57,82,83^ and to the adsorption isotherm^84^ and mesoscale confinement;^85^ we have also clarified the similarity and difference between solvation and adsorption.^30,31,86^ A model-free quantification of solvent–solvent or adsorbate–adsorbate interactions sheds light on the molecular basis of formulation processes.^84,87−89^

We will show that a universal theoretical framework can be applied to adsorption and absorption and that the sorbate–sorbate interaction plays a key role in understanding the functional shape of an isotherm. Similarities and differences between liquid and solid sorbents will be clarified (see Theory). We will demonstrate that an isotherm that includes the Langmuir,^22^ BET,^23,24^ and GAB^25−27^ models as its special cases can be derived directly from sorbate–sorbate interaction without assuming adsorption sites and layers. Furthermore, the existing isotherm models will be repurposed purely as convenient fitting functions without their claimed adsorption mechanisms (see Results and Discussion).

## Theory

### Statistical Thermodynamics of Adsorption

Consider a phase (denoted as *) consisting of a sorbent (species 1) and sorbate (species 2). The key to studying sorption is the concept of the excess number for the species *i*,
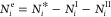
1We are considering, in eq 1, the entire system, with the superscript *, composed of the sorbate and sorbent in equilibrium, as well as the reference state with the superscripts I and II. The reference systems I and II are the sorbent interior and sorbate phases, respectively, in the absence of an interface. Note that there is no such thing as the “interfacial phase” as a separate entity. Rather, the presence of the interface is quantified by the difference between the system and the two reference systems.^30,31,80,84,90^

Adsorption (i.e., sorbates cannot penetrate the sorbent) and absorption (i.e., sorbates can move into the sorbent) are considered two subcategories of sorption.^91^ Therefore, we need to extend our previous paper on adsorption^84^ to incorporate absorption. To this end, we begin by summarizing our statistical thermodynamic foundation.^84^ To study surfaces (with the surface area *A*_S_) without any limitations on shape and porosity, we have generalized the Gibbs adsorption isotherm and statistical thermodynamics and derived^84^
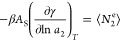
2using only the basic principles of partially open ensembles under the generalized Gibbs dividing surface condition,^84^
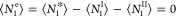
3applicable to any surface geometry, even in the presence of cavities and crevices. Note that ⟨⟩ denotes ensemble average. The location of the Gibbs dividing surface is specified with eq 3 by referring to component 1.

Understanding a sorption isotherm microscopically means explaining its functional shape (i.e., the IUPAC types) based on the underlying molecular interactions. Sorbate–sorbate interaction has been considered to play a key role in determining the shape of an isotherm.^9,92−95^ Recently, we have shown, via rigorous statistical thermodynamics,^84^ that adsorbate–adsorbate interaction can be quantified directly from an isotherm’s derivative, which is the key to classifying functional shapes; the activity (*a*_2_) dependence of the adsorbed quantity, ⟨*N*_2_^e^⟩, is related rigorously to the adsorbate–adsorbate number correlation, as^84^
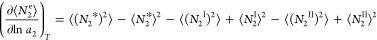
4

When applying eq 2 to adsorption, we ignore absorption, that is, *N*_2_^I^ = 0, and consider that the adsorbent is composed of species 1, which does not dissolve or evaporate into phase II, such that *N*_1_^II^ = 0. We postulate that the effect of an interface is confined within a finite distance from the surface, which we refer to as the subsystem (with volume *v*). Dividing the partially open ensemble into a local subsystem and a bulk adsorbate vapor system,^84,96^ we can rewrite eq 2 as
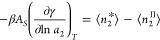
5ain terms of the difference in the adsorbate number between the interfacial subsystem ⟨*n*_2_^*^⟩ and the adsorbate subsystem ⟨*n*_2_^II^⟩ with the same volume *v*.^84^ (Here, the lower-case characters signify the numbers and volume pertaining to the local subsystem.) Our results, so far, have been general and without restrictions. From here onward, we shall consider the adsorption of vapor because of the wealth of applications and high-quality experimental data. Since vapor density is much lower than that of the adsorbates at the interface, we neglect ⟨*n*_2_^II^⟩; therefore,
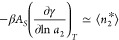
5b

Equation 4 can also be written using the local subsystems,^84^
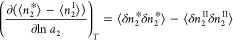
6awhere δ*n*_2_^*^ = *n*_2_^*^ – ⟨*n*_2_^*^⟩ and δ*n*_2_^II^ = *n*_2_^II^ – ⟨*n*_2_^II^⟩. Since the vapor-phase fluctuation is negligibly small, eq 6a leads to
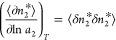
6bNote that ⟨δ*n*_2_^*^δ*n*_2_^*^⟩ is the sorbate–sorbate number correlation in the presence of the interface.

### Generalization to Absorption Isotherms

Now, we generalize eq 6a to the absorption isotherm. Our theoretical foundation is eq 4, which was derived under the generalized Gibbs dividing surface, eq 3. We again consider that the absorbent is composed of species 1, whose dissolution or evaporation into phase II is negligible, such that *N*_1_^II^ = 0. Just as in the case of adsorption, we postulate that the effect of the interface on the vapor side is confined within a certain distance, inside the volume *v*. Since there is also absorption into the absorbent, we divide *N*_2_^*^ and *N*_2_^II^ into
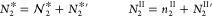
7where  is the number of absorbates in the volume *V*^I^ + *v* and *n*_2_^II^ in the volume *v* for the vapor reference system. The rest, *N*_2_^*′^ and *N*_2_^II′^, are the numbers of absorbates in the bulk. Because the effect of the interface on the side of phase II is confined within the volume *v*, *N*_2_^*′^ = *N*_2_^II′^.^84^ Following the same argument as eqs 30–34 of ref (84) in postulating that the correlation  is negligible compared to  and that ⟨δ*n*_2_^II^δ*N*_2_^II′^⟩ is negligible compared to ⟨δ*n*_2_^II^δ*n*_2_^II^⟩, we obtain
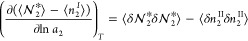
8aSince the vapor-phase fluctuation, ⟨δ*n*_2_^II^δ*n*_2_^II^⟩, is negligibly small, eq 8a leads to
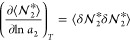
8bNote that  is the sorbate–sorbate number correlation in the presence of the sorbents.

Here, we have arrived at a significant conclusion: the adsorption isotherm (eq 6b) and absorption isotherm (eq 8b) have the identical functional form. The only difference is that eq 8b has taken absorption into account, whereas eq 6b did not.

### Understanding a Sorption Isotherm from Underlying Sorbate–Sorbate Interaction

We have established above that adsorption and absorption isotherms obey the same basic relationship. This means that adsorption and absorption can be analyzed in the same way, without any need for distinguishing between the two. We, therefore, adopt a common notation for a sorption isotherm. Using *n*_2_ as the quantity of sorption, we generalize eqs 6b and 8b into the following universal form
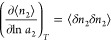
9a

Sorbate number fluctuation, ⟨δ*n*_2_δ*n*_2_⟩, determines the gradient of an isotherm when plotted against ln *a*_2_. Since how it increases is the main feature of an isotherm, the sorbate number fluctuation is the key to understanding the functional shape of an isotherm on a molecular basis.

Here, we introduce two alternative yet equivalent perspectives to facilitate the use of eq 9a for interpreting an isotherm based on sorbate–sorbate interaction. The first is the excess number of sorbate molecules around a probe sorbate molecule, *N*_22_, defined as^30,57,84^
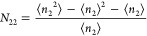
9bThe excess number represents the net number of additional sorbates that can be found around a probe sorbate compared to an expectation that a probe sorbate does not affect the spatial distribution of sorbates. *N*_22_ has a direct link to the gradient of an isotherm, as^84^
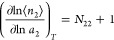
9cEquation 9c shows that the functional shape of an isotherm is characterized by sorbate–sorbate interaction quantified via the excess sorbate number.

The second perspective on sorbate–sorbate interaction information is the Kirkwood–Buff integral, *G*_22_, which is related to the excess number, as^30,57,84^
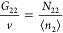
10a*G*_22_ is particularly useful because it has a microscopic interpretation via the sorbate–sorbate distribution function, *g*_22_(***r***) with ***r*** being the position vector, as^30,57,84^
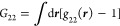
10bNote that *g*_22_(***r***) quantifies the sorbate–sorbate correlation in the presence of the sorbents. *G*_22_/*v* can be determined from the isotherm alone; to determine *G*_22_ itself, *v* must be measured experimentally because it cannot be quantified unless there is information about surface thickness.

The excess number and the Kirkwood–Buff integral depend not only on the direct interaction between a pair of sorbates itself but also on the interface and other sorbates mediating the interaction. *G*_22_ (and consequently *N*_22_) can either be positive or negative. When it is negative, the sorbates are excluded from the probe sorbate. Therefore, the excess number and the Kirkwood–Buff integral can handle both attractive and repulsive interactions. Defining an “interaction” exclusively as attractive and short-ranged has repeatedly brought confusion into the understanding of macromolecular solvation and conformational equilibria.^30,36,57,97^ Separate theoretical treatments were necessary for binding^98−100^ and exclusion^101,102^ for a long time with much confusion,^57,97^ until a unified treatment was introduced via the excess number and the Kirkwood–Buff integral.^30,36,57,97^ An excess number and the Kirkwood–Buff integral are therefore universal tools for solvation and sorption alike.

## Results and Discussion

### Sorption into Liquids and Solids across Deliquescence

Based on the excess number and the Kirkwood–Buff integral, we have established a universal language for the two different classes of phenomena, solvation and sorption (see Theory). Having a universal language is useful especially when a sorbent goes through deliquescence. One of the main questions in solvation is how a solute molecule changes the solution structure, or more specifically, the solvent–solvent interaction. For example, a long-standing mystery on the mechanism of strong, cooperative solubilization by hydrotropes was resolved by the enhanced hydrotrope–hydrotrope interaction by a solute molecule, quantified via the Kirkwood–Buff integral.^82,83,89,96,103^ This is analogous to a sudden, stepwise rise in the adsorption of water on mesoporous carbons attributed to the water cluster formation at the interface.^84^ Thus, how sorbate–sorbate interaction is mediated by a surface is analogous to how solvent–solvent interaction is mediated by a solute.^30,36^

Such an analogy between solvation and sorption necessitates an establishment of a theory of sorption for liquid sorbents and to compare it with solid sorbentes. As before, consider, for simplicity, a two-component solution consisting of a “sorbent” (species 1, solvent) and a “sorbate” (species 2, solute). In the liquid phase, it is natural to consider how the activity (or vapor pressure) of a species depends on the solution composition to probe interactions in solution. In doing so, we choose the solution composition as the variable and measure the change of activity. However, while this perspective is suitable for studying solvation, it is different from the one more convenient for sorption: taking the activity (or vapor pressure) of sorbate *a*_2_ as the variable to measure the solution composition, *N*_2_/*N*_1_. This is governed by its number fluctuation,^60,104^ as
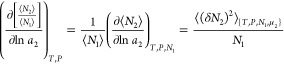
11

For clarity, throughout this paper, we denote the fixed ensemble parameters in {}. Equation 11 can be rewritten as
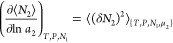
12

Sorption into liquid (eq 12) is analogous to sorption in/on solids (eqs 4 and 8a). Despite the apparent similarity, there is a subtle yet fundamental difference between liquid and solid sorbents: both *T* and *P* are kept constant in the sorption into liquids (eq 11), whereas *T* is the sole constant in the sorption in solids (eqs 4 and 8a). This difference comes directly from the Gibbs phase rule; a two-component solution in a single phase has one more degree of freedom than a (sorbent–sorbate) two-phase system.^30,31,86,89^ Consequently, the Gibbs dividing surface is introduced for solid sorbents, whereas there is no dividing surface for the liquid sorbent.

We must bear in mind that eq 12 presupposes a single-phased mixture of the sorbate and liquid sorbent. Therefore, if the sorbate and liquid sorbent do not mix and the sorbate (adsorbate, in this case) forms a film on the liquid sorbent surface, the system is in two phases and the adsorption theory for solid sorbates should be applied, instead of eq 12. On the other hand, when sorbates change sorbent–sorbent interaction, as in the case of swelling, we use eq 12. Thus, rather than the “liquid” and “solid” states of the sorbent, the degrees of freedom and the existence of the Gibbs dividing surface are the fundamental considerations when we have to choose between eqs 12, 4, and 8a as the basis of analysis. (In this paper, we will only analyze solid sorbents in a two-phase system with 2 degrees of freedom and liquid sorbents in a single-phase system with 3 degree of freedom.)

To understand the solution-phase interactions, we need to rewrite eq 11 using the local subsystems. Note that eq 11 is in a size-invariant form.^105^ Therefore, the relative fluctuation can be rewritten using a subsystem (still considered macroscopic), as^105^
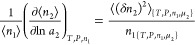
13

There is now an apparent similarity between eq 13 and the sorption isotherm expressed by local subsystems (eq 9a). This can be made clearer by rewriting eq 13 as
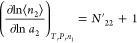
14a
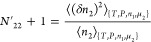
14b

Even though sorption in liquid, expressed via the subsystem (eq 14a), seems similar to sorption in solid (eq 9c), there is a fundamental difference between the two. The key is the difference in the ensembles adopted by the two. In liquids, not only the number of sorbates but also the volume of the {*T*, *P*, *n*_1_, μ_2_} subsystem fluctuates in eq 14b because *P*, instead of *v*, is kept constant.^105^ Such a sorbate–sorbate number correlation must be observed separately from the volume fluctuation. Converting the {*T*, *P*, *n*_1_, μ_2_} subsystem to a {*T*, *v*, *n*_1_, μ_2_} subsystem is necessary to single out the number fluctuation. This conversion is facilitated by our recent algebraic method based on the invariance of concentration fluctuation, in this case of *C*_2_ = *n*_2_/*n*_1_, as^105^
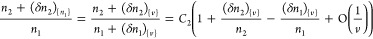
15awhich can be simplified as^105^
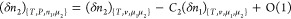
15b

Using eq 15b, eq 14a can be rewritten as
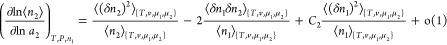
16

Equation 16 is the fundamental relationship for absorption in liquid sorbates. To clarify its physical meaning, we rewrite eq 16 in a manner analogous to sorption to solid sorbents, that is, eq 9c. To do so, let us use eq 9b again as the definition for the excess numbers, through which eq 16 becomes
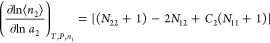
17awhere *N*_*ij*_ is defined (eq 9b) as the excess number of species *j* around species *i*. Using the Kirkwood–Buff integral, *N*_*ij*_ = *c*_*j*_*G*_*ij*_, eq 17a is transformed into a well-known expression in the Kirkwood–Buff theory of solutions^104,106^ that was used previously as the foundation for studying the water activity concept in liquid food^35^
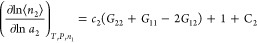
17bwhere *c*_2_ = *N*_2_/*V* is the concentration.

What, then, is the difference between liquid and solid sorbents? The crucial difference is the presence of sorbent–sorbent (*G*_11_) and sorbate–sorbent (*G*_12_) interactions, as can be seen by comparing eq 17a with eq 9a. Therefore, the following set of transformations (eq 18a) converts the isotherm for liquid sorbents (eq 16) to the one for solid sorbents (eq 9a)
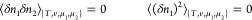
18aor equivalently
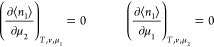
18bEquation 18a shows that a liquid sorbent transforms to a solid sorbent when the number of fluctuations involving sorbent molecules diminishes. From the solid side, the deliquescence transition introduces the fluctuations involving sorbent numbers and transforms the sorption theory for solids (eq 9a) to liquids (eq 17a). Since the number of fluctuations (eq 18a) distinguishes a solid sorbent and a liquid sorbent, a sorption theory for liquids cannot be applied directly to solids (Appendix A).

**Figure 1:**
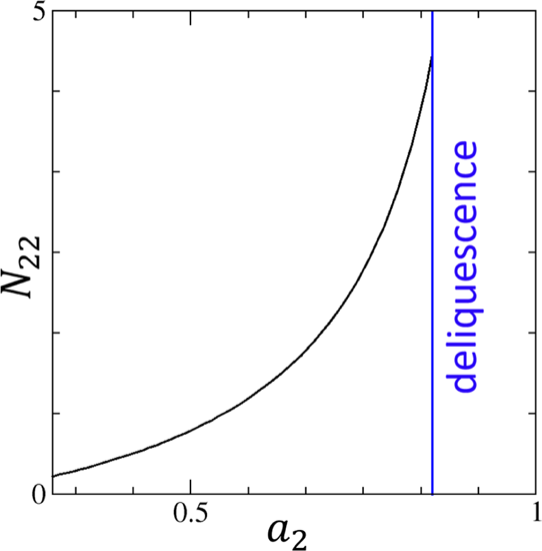
Water–water interaction, *N*_22_, underlying the moisture sorption isotherm of sucrose against the activity of water vapor, *a*_2_, calculated from the reported fit to the Oswin model (Appendix A) with the parameters *A* = 10.7708 and *B* = 0.8284, with an average error of 1.41% between *a*_2_ = 0.3 and 0.85^107^ (see Figure 429 and Table 1 therein). The blue line represents the deliquescence point of sucrose at *a*_2_ = 0.85.^71,72^

Thus, we have established a theory of sorption for solid and liquid sorbentes and clarified the transformation from one to another. Now, we compare solid versus liquid sorbents, taking amorphous sucrose as an example. On the solid side, we use the sorption isotherm at 25 °C as modeled by the empirical Oswin isotherm model between *a*_2_ = 0.3 and 0.85.^107^ Using the Oswin model as a fitting equation, the sorbate–sorbate (water–water) interaction can be calculated via statistical thermodynamics (eq 9c). See Appendix B for more details about this procedure. Figure 1 shows the change of water–water interaction with *a*_2_. The deliquescence point of sucrose is around *a*_2_ = 0.85.^71,72^ The discontinuity of *n*_2_ at this point^72^ is not captured by the Oswin model which can exhibit divergence only at *a*_2_ → 1 (see Appendix B). The increase of *N*_22_ with *a*_2_ shows that sorbates, despite their increase in quantity, do not behave like bulk water (in which case, *N*_22_ ≃ −1^108^).

Let us compare the moisture sorption isotherm of amorphous sucrose to that of aqueous sucrose solutions. We have analyzed the latter in detail in our previous papers^35,109^ based on the Norrish constants^2,110^ in the dilute sucrose region^35^ and on the activity model of Mathlouthi and Starzak^111^ in combination with the density data of the sucrose–water mixture^112^ in the concentrated sucrose region.^109^ The most important conclusion was that the sorbent–sorbent interaction is neither negligible nor minor, except in the concentrated region. This is demonstrated via the water–water, water–sugar, and sugar–sugar Kirkwood–Buff interactions (as in eq 17b); what makes the Norrish constant (essentially *G*_22_ + *G*_11_ – 2*G*_12_ in terms of the Kirkwood–Buff integrals) large and positive is the sorbent–sorbent (sugar–sugar) interaction, not the sorbate–sorbate (water–water) interaction.^35^ This illuminates a fundamental difference between absorption into a solid versus into a liquid: the mobility of the sorbent molecules.

### Connecting Sorbate–Sorbate Interaction to an Isotherm Model

#### Generalizing the Langmuir, BET, and GAB Models beyond Surface Adsorption onto a Plane

Here, we demonstrate that an isotherm model, which incorporates the Langmuir,^22^ BET,^23,24^ and GAB^25−27^ models as its special cases, can be derived without assuming adsorption sites and layers. Such a generalization will serve as the justification for the routine application of these models beyond planar multilayer adsorption^1−3,20,39−41,51^ with an additional benefit of increased freedom in the allowed range of parameters. Our foundation is the dependence of sorbate–sorbate interaction (quantified via *G*_22_/*v*) on sorbate activity, *a*_2_. (The sorbate–sorbate interaction, as explained in the Theory section, is under the influence of the sorbents.) Our starting point is the combination of eqs 9c and 10a, which yields
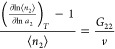
19aThis can be simplified as
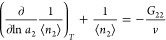
19bEquation 19b is a first-order differential equation. To solve this equation, we rewrite eq 19b as
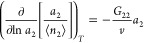
19cThe general solution of eq 19c is given as
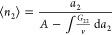
20where *A* is the constant of integration.

With the help of eq 20, a sorption isotherm model can be constructed directly from the dependence of sorbate–sorbate interaction on its activity. Here, we adopt the following simple relationship
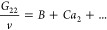
21awith *B* and *C* as constants. The coefficient *B* is the *a*_2_ → 0 limit of *G*_22_/*v*, which is also the *n*_2_ → 0 limit, as can be seen from eq 20. The coefficient *C* comes from sorbate–sorbate–sorbate correlation (Appendix C). When *C* = 0, there is no three-body contribution in *G*_22_. In general, an expansion up to the *n*th order of *a*_2_ must be considered in eq 21a if *n* body correlation between sorbates needs to be considered. Taking up to the first order of *a*_2_, we obtain the following isotherm from eq 21a via eq 20
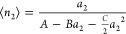
21bwith the following form suitable for determining the constants from experimental data
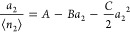
21c

A fitting equation similar to eq 21c has been used widely to determine the parameters for several sorption isotherm models, which are closely related to GAB and BET models.^23−27,113^ Such models have been classified as the “homogeneous sorption models” in the catalogue of sorption models by van den Berg and Bruyn.^13^ In this sense, eq 21b is considered to be a statistical thermodynamic generalization of the homogeneous sorption model.

Equation 21b was derived from the *a*_2_-dependence of the sorbate–sorbate interaction (eq 21a) without any assumptions on adsorption layers. It contains the Langmuir,^22^ BET,^23,24^ and GAB^25−27^ models as its special cases as we demonstrate below. The Langmuir isotherm (with the monolayer capacity *n*_m_ and the Langmuir constant, *K*_L_),
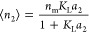
22acorresponds to the special case, *A* = 1/*n*_m_*K*_L_, *B* = −1/*n*_m_, and *C* = 0, of eq 21b. Consequently, the Kirkwood–Buff integral for the Langmuir model
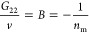
22bis a constant independent of activity. The negative sign of *G*_22_ shows that it is dominated by the constant excluded volume, *v*/*n*_m_, due to the repulsive interaction between sorbates. In contrast, the monolayer-based interpretation of eq 22b is simply to consider −1/*B* = *n*_m_ as the constant number of “binding sites”.

The dominance of the repulsive interaction is in contrast to the statement that there are no lateral interactions (i.e., adsorbates do not interact with one another) in the Langmuir model.^49,79,114^ Not only attractive but also repulsive interactions should be incorporated into the “sorbate–sorbate interaction” that determines the functional shape of an isotherm. *C* = 0 means that the Langmuir model neglects the contribution from higher-order correlations between sorbates. Thus, the Langmuir model can be derived from the dominance of the repulsive sorbate–sorbate interactions incorporated up to two-body correlation without using the monolayer adsorption on a planar interface.

Next, we turn to demonstrate that the BET and GAB models are the special cases within our isotherm, eq 21b. The GAB model, with the BET parameter *C*_B_ and the GAB parameter *K*_G_, has the following form
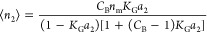
23ain which the BET model is its special case, *K*_G_ = 1. Comparing eq 23a with eq 21b shows that the GAB model is the special case of eq 21b with *A* = 1/*C*_B_*K*_G_*n*_m_, *B* = (2 – *C*_B_)/*C*_B_*n*_m_, and *C* = 2*K*_G_(*C*_B_ – 1)/*C*_B_*n*_m_. This leads to the following expression for the Kirkwood–Buff integral of the GAB model
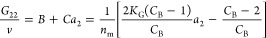
23bFrom eqs 23a and 23b, the excess number can also be expressed as
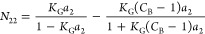
23c

Equation 23b shows that the sorbate–sorbate Kirkwood–Buff integral of the GAB model is a linear function of *a*_2_ and a special case of eq 21b. Equation 21b does not have restrictions on the range of values for *A*, *B*, and *C* introduced by the multilayer adsorption model and is considered to be a generalization of the GAB and BET models. Equation 21b was derived solely from an *a*_2_-dependence of *G*_22_, and incorporating up to the first order of *a*_2_ is equivalent to the presence of a three-body correlation between sorbates, which is independent of *a*_2_ (see Appendix C). This foundation is more general than the monolayer and multilayer adsorption mechanism assumed by the GAB model and serves not only as a justification of the widespread use of the GAB model beyond its original model assumptions but also to allow a wider range of values for the fitting parameters, *A*, *B*, and *C*. Moreover, the fitting at higher *a*_2_ may be refined, if necessary, by incorporating higher-order terms of *a*_2_ into the polynomial and consequently the multiple-body correlations between sorbates.

#### Sorbate–Sorbate Interaction Determines the Functional Shape of an Isotherm Regardless of the Fitting Models

Here, we show that the calculated sorbate–sorbate interaction is independent of isotherm models and their assumptions, even when multiple different models can fit an isotherm equally well. However, the limiting behavior of the isotherm at the *a*_2_ → 0 limit should also be considered, which must satisfy the condition imposed by Henry’s law.^115,116^

Such a consideration was inspired by an important recent review by Peleg,^20^ who raised questions on the monolayer concept for water. Peleg suggested that “isotherm’s shape alone does not contain enough information to uniquely identify and quantify the underlying sorption mechanisms”^20^ because multiple isotherm models, each assuming different adsorption mechanisms or none, can often fit an experimental isotherm equally well.^20,21,117^ Indeed, the purely empirical Peleg model^21^ can fit some experimental data as closely as the BET and GAB models.^20^ The Peleg model, with its four parameters, *A*_P_, *B*_P_, α_P_, and β_P_, has the following form
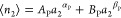
24Using eq 9c, we obtain the following expression for the sorbate–sorbate interaction
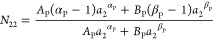
25Using eqs 24 and 25, the Kirkwood–Buff integral can be expressed as
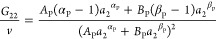
26

**Figure 2:**
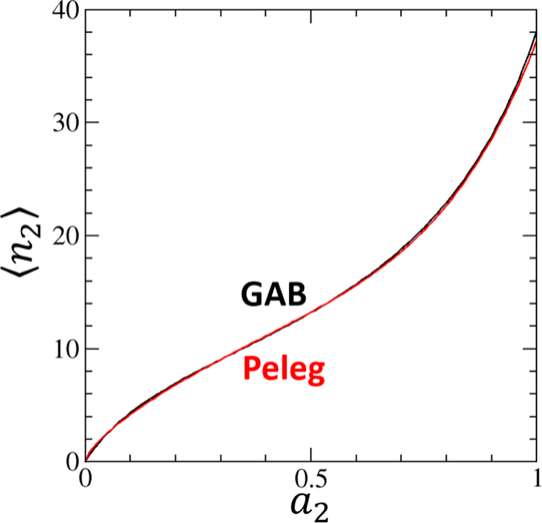
Moisture sorption isotherms of potato starch calculated from the GAB model (black line, eq 23c) and the Peleg model (red line, eq 25) using the fitting parameters provided by Peleg.^21^ The units of ⟨*n*_2_⟩ are % dry basis.

**Figure 3:**
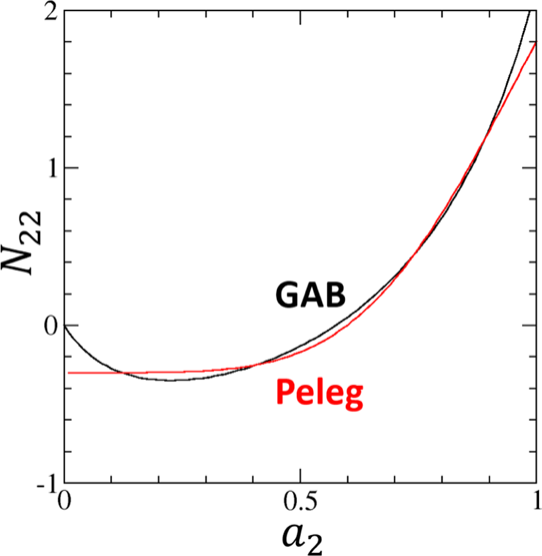
Comparison of water–water interaction expressed via the excess number, *N*_22_, calculated from the GAB model (black line, eq 23c) and the Peleg model (red line, eq 25) for the moisture sorption isotherm of potato starch using the fitting parameters provided by Peleg.^21^

Figure 2 shows the moisture sorption isotherm, ⟨*n*_2_⟩ against *a*_2_, of potato starch from the fitting using the GAB and Peleg models.^21^ Comparative goodness of fit by both models for the experimental isotherm data^21^ (Figure 2) leads to a good agreement of sorbate–sorbate (water–water) interaction *N*_22_ between the two models, except for *a*_2_ ≃ 0 (Figure 3). Note that Henry’s law imposes that the limiting behavior *N*_22_ → 0 must be satisfied at *a*_2_ → 0. This can be demonstrated by starting from a linear relationship with a constant *k*_H_, ⟨*n*_2_⟩ = *k*_H_*a*_2_, which reflects the proportionality between the sorbate quantity (⟨*n*_2_⟩) and the vapor pressure (*P* = *P*_0_*a*_2_, with *P*_0_ being the pressure at saturation). Substituting this linear relationship into eq 9c, we can prove that *N*_22_ = 0 in this linear region. Figure 3, therefore, shows that the GAB model satisfies this limiting behavior, but the Peleg model does not.

**Figure 4:**
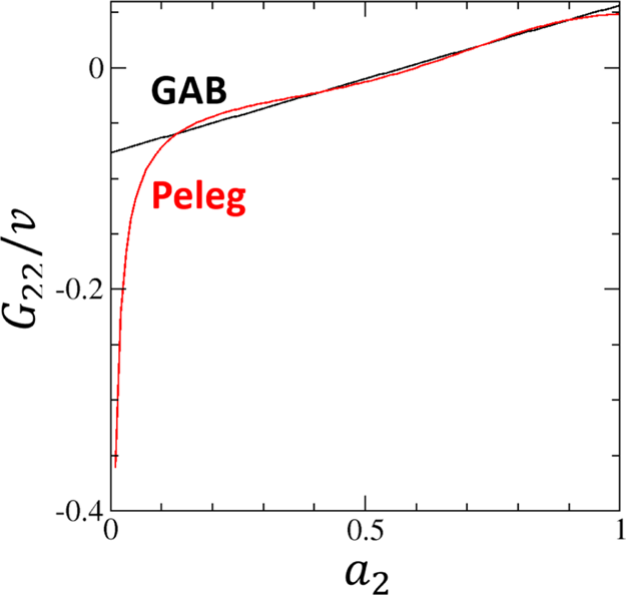
Comparison of water–water interaction expressed via the Kirkwood–Buff integral, *G*_22_/*v*, calculated from the GAB model (black line, eq 23b) and the Peleg model (red line, eq 26) for the moisture sorption isotherm of potato starch using the fitting parameters provided by Peleg.^21^ The units are (% dry basis)^−1^.

Despite the difference in the basic assumptions of the GAB and Peleg models, the underlying sorbate–sorbate interaction, expressed in terms of *G*_22_/*v*, is very close to one another, except, again at *a*_2_ → 0, where the Peleg model does not satisfy Henry’s law (Figure 4). Despite this, in most ranges of *a*_2_, sorbate–sorbate interactions calculated from the two very different models are very similar to one another. This is a demonstration of the universality of *N*_22_ and *G*_22_/*v*, regardless of the assumptions made in the fitting models.

#### Sorbate–Sorbent Interaction

What the “monolayer capacity” *n*_m_ calculated by the BET model means has been questioned.^20,118^ The “BET surface area”, a widely used measure of sorption, is calculated from *n*_m_ together with the adsorbate cross-sectional area and molar volume.^24^ However, a discrepancy between the “BET surface areas” for nitrogen and water has been reported widely.^20,118^ Such an inconsistency, arising from the application of the isotherm model beyond its limits, yet again motivates a general statistical thermodynamic approach based on the expansion of the Kirkwood–Buff integral (eq 21b).

We have already established the physical meaning of the parameters *B* and *C*. Here, we clarify the interpretation of the parameter *A*. To this end, let us start from the limiting behavior of eq 21b at *a*_2_ → 0,
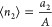
27

The activity *a*_2_ is defined as *a*_2_ = *P*/*P*^o^, where *P*^o^ is the saturation pressure of vapor. Using the ideal gas equation of the state, ⟨*n*_2_^II^⟩ = *Pv*/*RT* yields the number of vapor sorbates contained in volume *v*. Taking all together, eq 27 can be rewritten as
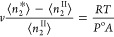
28Here, we have used the original expression, ⟨*n*_2_*⟩ – ⟨*n*_2_^II^⟩, instead of its abbreviation, *n*_2_, introduced in the Theory section. Noting that the left-hand side of eq 28 is the Kirkwood–Buff integral between the sorbate surface and sorbent, *G*_*s*2_, we obtain
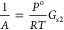
29Thus, the parameter *A* is related to the sorbate–sorbent Kirkwood–Buff integral at the *a*_2_ → 0 limit.

The BET model is the special case of eq 29, in which *A* = 1/*n*_m_*C*_B_. Therefore, *G*_*s*2_ can be expressed in terms of the BET parameters as
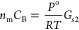
30that is, the product of the monolayer capacity, *n*_m_, and the BET parameter, *C*_B_. To determine the BET parameters, the gradient and intercept of the linearized plot
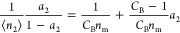
31are combined to determine *n*_m_ and *C*_B_.^49,79^ From eq 30, *G*_*s*2_ is related to the intercept of this plot. The independent determination of *n*_m_ and *C*_B_ assumes *A* = 1/*C*_B_*n*_m_, *B* = (2 – *C*_B_)/*C*_B_*n*_m_, and *C* = 2(*C*_B_ – 1)/*C*_B_*n*_m_ in eq 21b, which leads to *C* = 2(*A* – *B*), meaning that the three-body sorbate interaction is expressed by sorbate–sorbent and sorbate–sorbate interactions. We emphasize here that the parameters *n*_m_ and *C*_B_ are determined by both the sorbate–sorbent and sorbate–sorbate Kirkwood–Buff integrals. Therefore, from a Kirkwood–Buff perspective, neither *n*_m_ nor *C*_B_ corresponds purely to the sorbate–sorbent and sorbate–sorbate interaction. Since eq 29 does not involve any assumptions on the mode of sorption (such as adsorption, absorption, and surface geometry), it can attribute a physical meaning to the parameter *A* in terms of sorbate–sorbent interaction.

#### Extending the Fluctuation Theory of Sorption

We have thus demonstrated that our statistical thermodynamic approach,^84^ when applied to an adsorption model, can reveal its underlying molecular interactions. (A further example, the Fractal FHH model,^14−16^ is examined in Appendix D.) This is an extension of our previous approach to sorption, clarifying the molecular interactions underlying empirical models that may be different from what they had originally assumed.^36,88,119,120^

In applying our general statistical thermodynamic theory, we have focused on relatively simple sorption isotherms that can be modeled via expanding the Kirkwood–Buff integral around *a*_2_ → 0, taking up to sorbate three-body interactions that are, of course, influenced by the presence of the sorbent. The sorbent surface structure has been incorporated only as an average in *n*_2_. How surface heterogeneity affects sorption isotherms, a question particularly important in microporous and mesoporous interfaces,^24,93^ will be addressed in a forthcoming paper. This requires an explicit consideration of the partition function underlying eq 8b.^84^ Interpreting the temperature dependence of sorption is also presented in a forthcoming paper.

## Conclusions

Attempting to understand the sorption mechanism by fitting an isotherm model to an experiment may end up inconclusive when multiple isotherm models, with different assumptions on sorption mechanisms,^13−18^ fit an experimental isotherm equally well.^20^ Some isotherm models (such as BET and GAB models^23−27,32−34^) are used to fit experimental systems beyond their underlying assumptions,^20^ and a discrepancy between the assumption (planar multilayer adsorption) and reality (often absorption with swelling) has been widely recognized in the literature.^20,51,118^

Such difficulties can only be overcome by a universal approach to determining the sorption mechanism directly from an experimental isotherm. We have shown that sorbate–sorbate interaction, the key to understanding the functional shape (type) of an isotherm,^9,92−95^ can be quantified directly from an isotherm. We have constructed a theory applicable universally to adsorption and absorption,^29^ solid and liquid sorbents,^30,31^ and vapor and liquid sorbates, making it possible to analyze an isotherm from both sides of the deliquescence transition.

We have demonstrated that different isotherm models fitting to a single data do not pose any difficulties in interpretation; they simply lead to the same sorbate–sorbate interaction. Based solely on the dependence of the sorbate–sorbate Kirkwood–Buff integral on activity, we have constructed an isotherm, which contains the Langmuir and the GAB models as its special cases, directly from the Kirkwood–Buff integrals without introducing any assumptions on adsorption layers. Unlike adsorption models, our theory is model-free and is founded on the principles of statistical thermodynamics, according to which the sorbate–sorbent and sorbate–sorbate Kirkwood–Buff integrals play a key role in elucidating the microscopic mechanism underlying an isotherm. This theory will be extended to cover the temperature dependence of sorption in a forthcoming paper.

## Appendix A

In the statistical thermodynamic isotherm theory of Zimm^60^ and Zimm and Lundberg,^61^ only sorbate–sorbate interaction is present. Their theory,

A.1

was derived from the Kirkwood–Buff theory of solutions, namely, eq 17a, in combination with the Gibbs–Duhem equation (note that eq 10 of ref (60) has an incorrect negative sign in front of ).^60^ The presence of constants *T* and *P* in eq A.1 is different from the isotherm with a solid-state sorbate (eq 9a), in which only *T* is kept constant. In the derivation of eq A.1 from eq 17a, *G*_11_ and *G*_12_ were converted to κ_*T*_ (the isothermal compressibility) and *V*_1_ (partial molar volumes of the species 1)^60^ via the Gibbs–Duhem equation. This means that (i) eq A.1 presupposes a single-phase solution^31,84^ and (ii) *G*_12_ and *G*_22_ do not appear in the Zimm theory but are contained implicitly in *V*_1_ and κ_*T*_. Therefore, the Zimm theory of adsorption is valid only for liquid sorbents, despite many cases of its applications to solid sorbents.

## Appendix B

The Oswin model^19^ is a purely empirical relationship based on the Pearson Type XII distribution.^121^ Hence, it does not assume any underlying mechanism of adsorption on a molecular basis. This model, with the parameters *A* and *B*, can be expressed as

B.1

Using eq 9c, we obtain the following expression for sorbate–sorbate interaction

B.2

The divergence of ⟨*n*_2_⟩ and *N*_22_, according to the Oswin model, takes place only at *a*_2_ → 1.

## Appendix C

Here, we show the physical interpretation of the parameter *C* in eq 21a, expressed as

C.1

where the Kirkwood–Buff integral is expressed using the inhomogeneous ensemble, *n*_22_, which is the ensemble average of *n*_2_ in the system with a fixed molecule of species 2, as

C.2

Combining eqs C.1 and C.2

C.3

The first term of eq C.3 can be evaluated, using Equation (B10) of ref (82) in the absence of species 1, as

C.4

Note that ⟨δ*n*_2_δ*n*_2_⟩_2_ – ⟨δ*n*_2_δ*n*_2_⟩ signifies the increase in sorbate–sorbate correlation caused by the presence of a fixed sorbate molecule. Equation C.4 therefore represents the three-body correlation involving sorbates. The second term of eq C.3, using the definitions of the Kirkwood–Buff integral (eqs C.2, 9b, and 10a), can be expressed as

C.5

Combining eqs C.4 and C.5, we obtain

C.6

Equation C.6 shows that *C* is the difference between three-body and two-body correlations. The expansion up to the first order of *a*_2_ is possible in eq 21a when *C* is a constant independent of *a*_2_.

## Appendix D

Here, we calculate the sorbate–sorbate interaction underlying the Fractal FHH model.^14−16^ This model, which has the two constants, *n*_m_ and *D*, is expressed as

D.1

where *D*, according to this model, is the fractal dimension between 2 and 3 (embedding system). To substitute this equation into eq 9c we rewrite eq D.1 as

D.2

Then the fluctuation theory yields

D.3

Equation D.3 is a self-similar relationship.

Let us clarify the physical picture underlying eq D.3. To do so, let us note that eq D.1 has been justified based on a macroscopic approach and a microscopic lattice model.^15^ Both justifications assume that the structure of adsorbates is identical to that of the bulk solvent.^15^ For a bulk solvent, the Kirkwood–Buff theory yields *N*_22_ ≃ −1, which is equivalent to the Gurvitsch rule for adsorbate density.^108^ This is satisfied only at *D* = 3 (eq D.3) when the adsorbate dimension is the same as that of the embedding system. At *D* = 2, when the original FHH was derived, the fluctuation diverges with the increase of *a*_2_ or ⟨*n*_2_⟩, contradictory to that of the bulk solvent as assumed by the FHH model. Thus, the “fractal dimension” *D*(14−16,42,122,123) is, in fact, a parameter governing how adsorbate–adsorbate interaction, *N*_22_, depends on the adsorption potential, −*RT* ln *a*_2_.
